# Past and present: changes in the Odonata fauna of small lowland watercourses

**DOI:** 10.3897/BDJ.11.e107919

**Published:** 2023-10-17

**Authors:** Kálmán Szanyi, István Grigorszky, László J Szabó, György Dévai

**Affiliations:** 1 Department of Hydrobiology, Faculty of Science and Technology, University of Debrecen, Debrecen, Hungary Department of Hydrobiology, Faculty of Science and Technology, University of Debrecen Debrecen Hungary; 2 Juhász-Nagy Pál Doctoral School of Biology and Environmental Sciences, University of Debrecen, Debrecen, Hungary Juhász-Nagy Pál Doctoral School of Biology and Environmental Sciences, University of Debrecen Debrecen Hungary

**Keywords:** intermittent, climate change, drought, biodiversity, secondary habitat, dragonfly

## Abstract

Small lowland watercourses, strongly exposed to anthropogenic activities and climate change, have received negligible odonatological attention. This study provides a revised checklist of three typical lowland small watercourses (Kállai-főfolyás, Konyári-Kálló and Ölyvös) within the Pannonian Lowland and presents the changes in their diversity over the past decades. Results revealed a significant biodiversity loss, with a 31.6% decline in Odonata fauna over the last 53 years. The upper and middle sections degraded the most, where the habitats have dried out or become intermittent. However, a diverse Odonata assemblage (1,277 individuals of 27 species) was observed at the 14 sampling sites of the three watercourses, containing protected and sensitive species (*Somatochloraflavomaculata*, *Orthetrumbrunneum*, *Aeshnaisoceles*, *Libellulafulva*). However, the low abundance of larval and exuvial forms (59 individuals of 13 species) suggests that the majority of the observed adults were developed in other watercourses. While recolonisation from nearby habitats is still possible, a parallel degradation of adjecent waterbodies could lead to an irreversible biodiversity loss.

## Introduction

Wetlands are known as one of the most species-rich freshwater habitats, playing a crucial role in the maintenance of biodiversity (Giller and Malmqvist 2000, Brysiewicz et al. 2022). Small watercourses belonging to wetlands have unique habitat characteristics that are not found elsewhere (Ferraira et al. 2022). Their number and total length are underestimated, as they constitute over 70% of the total watercourse length in either European (Kristensen and Globevnik 2014) or global catchments (Downing et al. 2012, Ferraira et al. 2022). Despite their significant contribution to hydrographic networks, minimal attention has been devoted to conducting hydroecological surveys on them. Consequently, the majority of them remain unprotected and absent from most of the official bioassessment programmes (Ferraira et al. 2022).

Ecological status of small watercourses is gaining awareness recently, due to the increasing effects of the climate change (Williams et al. 2004, Downing et al. 2006, Verdonschot et al. 2011, Bartout et al. 2015, Biggs et al. 2017). However, most studies evaluate these effects only in mountainous streams (Principe et al. 2007, Lewin et al. 2013, Brysiewicz et al. 2022), whereas small watercourses in lowland areas are still under-investigated (Somlyai et al. 2019, Brysiewicz et al. 2022). Nonetheless, small watercourses in lowlands provide special habitat conditions with their flat nature: there is almost no channel slope, low depth, prevalence of soft grounds, extensive thickets of aquatic macrophytes and high summer temperatures. These watercourses are increasingly exposed to climatic and anthropogenic effects, since most of them flow through agricultural fields almost in their entire length. Their catchment areas are significantly impacted by agricultural and forestry utilisation (Chesterton 2009, Elosegi et al. 2010, Maynou et al. 2017, Somlyai et al. 2019); water abstractions also frequently occur from them, mainly for irrigation and for filling fishponds. Due to the combination of these negative factors, many of these small watercourses have nearly or completely dried out over the past few decades (Georgakakos and Kavvas 1987, Gomi et al. 2002, Schertzer et al. 2002, Lake 2003, Rigby and Porporato 2010, Somlyai et al. 2019). However, more water would be required in their beds to counteract the further effects of climate change, meet the growing societal water demands and, not least, maintain their biodiversity.

For the investigation of long-term changes in water quality and the ecological status of a watercourse, macroinvertebrates serve as ideal indicators (Tripole et al. 2008, Lewin et al. 2013, Worthington et al. 2015, Brysiewicz et al. 2022), due to their sensitivity to oxygen concentration, water chemistry (Saloom and Duncan 2005), food availability (Cross et al. 2006) and changes in habitat structure (Steinman et al. 2003, Tripole et al. 2008, Lewin et al. 2013, Brysiewicz et al. 2022). Amongst them, dragonflies, as a “flagship” or “umbrella” group (Chovanec et al. 2002, Bried et al. 2007, Oertli 2008, Balzan 2012, Maynou et al. 2017), excel (Samways et al. 2010, da Silva Monteiro Júnior et al. 2013, Bried and Samways 2015, Bouhala et al. 2019, Nagy et al. 2019), with their high diversity, complex life history, rapid development, broad distribution, relatively long life and essential role in food webs (Clark and Samways 1996, Corbet 1999, Briers and Biggs 2003, Córdoba-Aguilar 2008, Catlin 2009, Remsburg and Turner 2009, Simaika and Samways 2011, de Oliveira-Junior et al. 2015, Bouhala et al. 2019, Nagy et al. 2019). Dragonflies inhabit intrinsically patchy habitats as their larvae are restricted to freshwater ecosystems, while their adults mostly stay near breeding sites (Maynou et al. 2017). Consequently, healthy and stable aquatic habitats are essential for their larvae and resource-rich terrestrial habitats are vital for adult maturation, feeding, resting and mating (Raebel et al. 2012, Nagy et al. 2019), emphasising the significance of dispersal in their ecology (Maynou et al. 2017).

Adult dragonflies locate aquatic biotopes using visual cues and assess their quality to minimise the risks of dispersal (Wildermuth 1998). However, habitat fragmentation caused by the degradation of small watercourses inhibits their movement between habitat patches, increasing their mortality during dispersal (Maynou et al. 2017). It leads to biodiversity loss and aquatic ecosystem homogenisation towards generalist taxa (Couceiro et al. 2011, de Oliveira-Junior et al. 2015). Despite being mostly human-made habitats, lowland small watercourses are important "secondary biodiversity hotspots" (Dolny et al. 2007, Harabis and Dolny 2012) highlighting the need to preserve these small habitat patches serving as stepping stones for dragonflies and other wetland-related organisms (Maynou et al. 2017).

In this study, we investigated the long-term changes in the diversity of Odonata assemblages living in small lowland watercourses. The revised checklist of the Odonata fauna of three typical small watercourses of the Pannonian Lowland was provided and was compared to the fauna described from there decades before.

## Materials and Methods

### Sampling area

Collections were carried out in three watercourses: Kállai-főfolyás (Kf), Konyári-Kálló (KK), and Ölyvös (Ö), located in Nyírség (Kf and partly KK) and Berettyó–Körös-vidék (Ö and partly KK) regions. These regions are dry areas (aridity index ~ 1.20) with limited water availability and poor run-off. Nyírség Region is dominated by sandy soils, featuring high infiltration rates and hydraulic conductivity, moderate field capacity and reduced water retention. Contrarily, the Berettyó–Körös-vidék Region is characterised by meadow and saline soils (Dövényi 2010). Thus, sediments of the watercourses are predominantly composed of fine sand, supplemented with mud and clay.

Nyírség and Berettyó–Körös-vidék regions are situated in the eastern part of the Pannonian Lowland, which has a continental climate. During the 20^th^ century, precipitation frequency of the area has reduced (Bartholy and Pongracz 2005), while its mean annual temperature increased by ~ 1^o^C (Bartholy et al. 2014). These trends became more intensive in the first period of the 21^st^ century (Bartholy et al. 2014) and the most arid season in the region has shifted from winter to late summer (Bartholy et al. 2014). Consequently, these climatic changes have disrupted the water regime of lowland small watercourses in this region, leading to the drying out of its formerly permanent watercourses (Szabó et al. 2018, B-Béres et al. 2019, Somlyai et al. 2019).

These watercourses, considered as lowland small watercourses according to the ecological waterbody typology (Dévai 1976, Dévai 1997, Dévai et al. 2005), have astatic character since they usually dry out for a longer time in almost their entire length. Flowing through agricultural fields and settlements, all three watercourses are significantly impacted by anthropogenic activities. We surveyed all habitat types within the watercourses, selecting 14 sampling sites, based on considerations of their length, altitude zones and water permanency (Table 1).

### Sampling methods

Studies of Dévai and Miskolczi (1999), Dévai and Miskolczi (2009), Dévai and Miskolczi (2011) and Viski et al. (2013) provided detailed information regarding the former Odonata fauna of the selected sampling sites (Table 2).

Recent field investigations were carried out in 2019 and 2021, from April to October. In 2019, only the sampling sites of Kállai-főfolyás were surveyed (Dévai et al. 2021), while the research extended to all sampling sites in 2021 (Table 2).

As previous surveys were made mainly based on adults, the current study also placed a greater emphasis on their examination. Seven samplings were conducted at each sampling site, covering the entire phenology of adult dragonflies. Sampling of adults were carried out with a standard butterfly net (with 1 mm mesh size, 500 mm depth, 260 mm wide frame), supplemented with observations using Carena 8x22 binoculars. Additionally, three larval samplings were conducted at each site to obtain more information about the fauna. Larval samplings were made with a standard kick-net (250 ×250× 300 mm, Ø 1 mm net), focusing on the foreshores, macrovegetation and surface of sediment.

Identification of the caught specimens was made, based on the keys of Dijkstra and Lewington (2006) and Ambrus et al. (2018) and the nomenclature of Ambrus et al. (2018) was followed.

### Data analysis

To characterise the fauna, a summarised checklist was made combining published and non-published distribution data.

Sampling efforts varied across different years and sites; thus, the comparison of former and recent assemblages was made in case of Konyári-Kálló, since its sampling sites showed similar sampling efforts. Former investigations were carried out in 2008-2009 and 2011-2012, which were repeated in 2021 (Table 2).

To evaluate the diversity of the different sampling sites, watercourses, altitude zones and habitat types, Shannon-Wiener and Simpson 1-D diversity indices were calculated. Additionally, a new Odonata Conservation Index (OCI) was employed to evaluate the conservation values of the sampling sites, watercourses and habitat types. OCI was established, based on Grasshopper Conservation Index (GCI) introduced by Matenaar et al. (2015) and Szanyi et al. (2021) and Caddisfly Conservation Index (CCI) published by Szanyi et al. (2022). OCI integrates dispersal ability, local rarity and vulnerability of species.

Dispersal ability categories – high (= 1), good (= 2), moderate (= 3) and low (= 4) – were determined, based on the study of Harabis and Dolny (2012). Local rarity was measured upon actual relative frequencies (RF%) of species occurring at the studied 14 sites, classified as common (= 1; RF% > 0.038841), frequent (= 2; RF% = 0.014797-0.038841), occasional (= 3; RF% = 0.00493-0.014797) and rare (= 4; RF% < 0.00493). Vulnerability of species was categorised as not threatened (= 1), protected or threatened (= 2), vulnerable (= 3) and endangered (= 4), based on the Hungarian Red List and the conservational status of Odonata species in Hungary (Ambrus et al. 2018). These three parameters were summed and divided by 12 (maximum value) to obtain an OCI value between 0.25 and 1. The OCI value of a sampling site was determined as the sum of the values of all its species. A Standardised Odonata Conservation Index (OCI') was also calculated, by dividing OCI by the number of species at the given site. Opposed to OCI, OCI' values are not influenced by species-richness (Matenaar et al. 2015, Szanyi et al. 2021, Szanyi et al. 2022).

## Results

### Recent Odonata fauna of the studied watercourses

In 2019 and 2021, a total of 1,277 individuals of 27 Odonata species were caught at the 14 sampling sites of the three watercourses (Table 3). A total of 16 Anisoptera and 11 Zygoptera species were detected at the studied sites (Ambrus et al. 2018). Kállai-főfolyás was the most species-rich watercourse with 25 species, followed by Ölyvös (20) and the poorest assemblage belonged to Konyári-Kálló (17). The majority of the collected species are characteristic for standing waters and slow-flowing small watercourses with rich macrovegetation and lowland character. It is especially true for the most abundant species, which were *Coenagrionpuella* (364), *Ischnuraelegans* (153), *Sympetrumsanguineum* (125) and *Calopteryxsplendens* (74). *C.puella* constituted 28.5% of the collected individuals. Beside the common and widespread species, the presence of several protected and sensitive species was observed. Amongst them, *Somatochloraflavomaculata*, classified as a vulnerable species in Hungary according to the latest Hungarian Red List (Jakab 2013, Ambrus et al. 2018), was detected in one of the studied watercourses (Kf). The threatened *Orthetrumbrunneum* occurred in two (Kf, Ö) out of the three watercourses. Additionally, *Aeshnaisoceles* and *Libellulafulva* were also recorded, these species being protected in Hungary (Jakab 2013, Ambrus et al. 2018).

### Assemblages of the studied sites

Comparison of the studied sampling sites, focusing on their recent Odonata assemblages, was carried out using the dataset of 2021, involving 811 individuals of 27 species (Table 4, Suppl. material 1).

Highest species-richness was found at the Kf3 (20) site, followed by Kf2, Ö3 (14 species each) and Ö1 (12) sites, while the most abundant assemblages were observed at Kf3 (124), KK4 (97), Ö3 (89) and KK6 (87) sites (Suppl. material 1). Three (KK1, KK2, Kf1) of the 14 sampling sites dried out completely during the entire survey, resulting in the absence of Odonata species in those locations. These dried sampling sites were located in the upper sections of Konyári-Kálló and Kállai-főfolyás watercourses.

Studied sampling sites were characterised with several diversity indices (Shannon-Wiener, Simpson, OCI, OCI’). Highest values of Shannon-Wiener index, sensitive for rare species, are produced by Kf3, Ö3, Ö1 and Kf2 sites. Contrarily, the Simpson diversity index, sensitive for the abundance of dominant species, gave the highest numbers to Ö3, Kf3, Ö1 and Ö2 sites. Both indices showed that the most valuable and diverse assemblages lived at the most species-rich sites, with the exception of the Ö2 site, where only eight species occurred. Values of Odonata Conservation Index mostly supported the ranking of the Shannon-Wiener diversity index, highlighting the assemblages of Kf3, Kf2, Ö3 and Ö1 sites.

As these indices strongly depend on the species-richness of a given habitat, OCI' values were calculated to mitigate this bias. According to OCI', the KK5 site, despite its relatively low species and individual number, proved to be one of the most diverse and valuable sites, alongside KF2, Kf3 and Ö1.

Between Kállai-főfolyás and Ölyvös, there were no large differences considering both species-richness, diversity (SW, Simpson) and conservation (OCI, OCI') indices and they showed higher values than those shown by Konyári-Kálló (Table 4). The number of differential species was the highest in Kállai-főfolyás (*Erythrommanajas*, *A.mixta*, *S.flavomaculata*, *Crocothemiserythraea*, *O.coerulescens*), while just a few differential species could be found in Ölyvös (*Chalcolestesparvidens*, *Brachytronpratense*) and Konyári-Kálló (*S.meridionalis*).

Sites located in the upper sections of the watercourses dried out completely, with the exception of the Ö1 sampling site. Thus, in the zonal comparisons, the upper section was represented only by the assemblage of the Ö1 site. There were no significant differences in the Simpson and Shannon-Wiener diversity values of the assemblages of the different sections. Despite OCI values showing outstanding differences amongst sections, underlining the high diversity of the middle section, OCI’ numbers did not support this. The middle section of the watercourses hosted the majority of species with high local rarity (*A.mixta*, *S.flavomaculata*, *S.meridionalis*, *C.erythraea*, *O.coerulescens*), but *B.pratense* were strongly related to the upper section and *A.isoceles* were found only at sites of the lower section.

Regarding the assemblages of the intermittent and permanent sites, neither species-richness nor diversity indices (SW, Simpson, OCI, OCI’) showed significant differences. Differential species occurred at sites with both permanent (*S.fusca*, *E.najas*, *A.isoceles*, *A.mixta*, *S.striolatum*) and intermittent (*C.parvidens*, *B.pratense*, *S.flavomaculata*, *S.meridionalis*, *C.erythrea*, *L.depressa*) water coverage.

### Larvae of the watercourses

Almost all dragonflies found at the sampling sites were in their adult stage. In 2021, only 59 individuals of 13 species were collected in larval or exuvial form (Table 5). Larvae of six species (*C.splendens*, *C.pulchellum*, *I.pumilio*, *S.meridionale*, *S.sanguineum* and *S.striolatum*) occurred only in sections with permanent water coverage. Contrarily, larvae of most anisopterans, except *Anaximperator* and species belonging to genus *Sympetrum*, were found only at sites with intermittent water condition. *S.meridionalis* was solely observed in its exuvial form throughout the given year.

### Changes in the Odonata assemblages of the studied watercourses

Detailed faunistic data were available about former Odonata assemblages of the studied watercourses. Earliest investigations were carried out in 1968 (Dévai and Miskolczi 2011), while more recent ones were implemented in 2012 (Viski et al. 2013) (Table 2). During the previous surveys, all sampling sites were characterised by permanent water coverage. A total of 38 Odonata species were recorded in the three watercourses as a result of former samplings (Table 3). The highest number of species was observed in Konyári-Kálló (34), but Ölyvös (27) and Kállai-főfolyás (20) also had diverse assemblages. The former checklist contained 24 Anisoptera and 14 Zygoptera species. Several vulnerable and sensitive species occurred in the watercourses; species of *C.ornatum*, *S.flavomaculata*, *S.pedemontanum* and *S.depressiusculum* are categorised as vulnerable, while *O.brunneum* is considered as threatened in the recent Hungarian Red List (Jakab 2013, Ambrus et al. 2018). Additionally, species of *A.isoceles*, *Gomphusvulgatissimus* and *L.fulva* are protected in Hungary (Jakab 2013, Ambrus et al. 2018) and *S.metallica* formerly belonged to the potentially endangered category (Ambrus et al. 2018). Several species from these have not been found recently: *C.ornatum*, *G.vulgatissimus*, *S.metallica*, *S.depressiusculum* and *S.pedemontanum*. However, the decline in biodiversity of the watercourses was not limited to these endangered species; a significant loss of fauna has been observed over the past half century, as 31.6% of their species disappeared (*Lestessponsa*, *L.virens*, *C.ornatum*, *Enallagmacyathigerum*, *G.vulgatissimus*, *S.metallica*, *L.quadrimaculata*, *O.cancellatum*, *S.depressiusculum*, *S.flaveolum*, *S.pedemontanum* and *S.vulgatum*). Most of these species are strongly related to habitats with permanent water coverage and moderate flow velocity. In the recent fauna, most of the remaining species are more generalist, common, drought-tolerant or possess high dispersal abilities.

Almost 50% of Odonata diversity have been lost within a decade in Konyári-Kálló, since 34 species occurred at seven sampling sites between 2008-2009 and 2011-2012, while in 2021, only 17 species were found (Table 6). Formerly, its local fauna consisted of 21 Anisoptera and 14 Zygoptera species, which have reduced to 10 Anisoptera and seven Zygoptera species. From the disappeared 16 species, 12 were anisopteran (*A.mixta*, *A.imperator*, *B.pratense*, *G.vulgatissimus*, *S.metallica*, *L.quadrimaculata*, *O.brunneum*, *O.cancellatum*, *O.coerulescens*, *S.depressiusculum*, *S.striolatum* and *S.vulgatum*), while four were zygopteran (*L.sponsa*, *L.virens*, *C.ornatum* and *E.cyathigerum*). Formerly, seven of the protected and/or vulnerable species of the Hungarian fauna were reported from the watercourse (*A.isoceles*, *L.fulva*, *C.ornatum*, *G.vulgatissimus*, *S.metallica*, *O.brunneum* and *S.depressiusculum*), but five of them have disappeared since then (*C.ornatum*, *G.vulgatissimus*, *S.metallica*, *O.brunneum* and *S.depressiusculum*). Additionally, the abundance of *L.fulva* notably decreased (RF% in 2008-2009, 2011-2012 = 12.63; RF% in 2021 = 0.31). Species sensitive to drought and/or a decrease in water flow velocity also disappeared (*C.ornatum*, *B.pratense*, *G.vulgatissimus*, *O.brunneum*, *O.coerulescens*, *S.depressiusculum* and *S.vulgatum*) (Dijkstra and Lewington 2006, Ambrus et al. 2018).

The former diversity of local Odonata assemblages was also higher (Table 6), characterised by more balanced species composition. Dominant and subdominant species included vulnerable species (*L.fulva*) and species preferring higher current velocities (*C.splendens*). Contrarily, the present fauna is dominated by two common, widespread and pioneer species (*C.puella* and *S.sanguineum*) (Ambrus et al. 2018), from which *C.puella* showed especially high relative frequency (RF% = 49.07).

Both species-richness and diversity values of all sampling sites have decreased significantly over the last decades (Table 6). Upper and middle sections of the watercourse degraded the most. Sampling sites of upper section (KK1, KK2) have become completely dry, resulting in the loss of their entire fauna. Sites of the middle section have also strongly degraded. The species number reduced by 76.92% at the KK5 site, while these reductions were 66.66% and 55% in the case of KK3 and KK4 sites, respectively. Comparatively smaller decreases were observed in the Odonata assemblages of the lower section (30.77% in the KK7 and 26.66% in the KK6 site). Formerly, lower section sites were amongst the least species-rich and diverse sites of the watercourse, while recently, these produce one of the highest diversity values and species numbers.

Development of the observed adults in the watercourse can be proved by the presence of their larvae and exuviae. In the previous survey, 17 species were collected at the sampling sites in larval or exuvial form (Table 7). The majority of these species (12) were collected from the upper section of Konyári-Kálló, but almost similar species-richness was found in all sections. Recently, only six species were found in larval or exuvial form. Although larvae and exuviae of two formerly unrecorded species (*C.puella* and *S.meridionale*) were observed during the present survey, larvae of 13 species (*C.parvidens*, *Lestesbarbarus*, *S.fusca*, *C.splendens*, *Platycnemispennipes*, *E.viridulum*, *A.mixta*, *A.isoceles*, *S.metallica*, *L.fulva*, *O.albistylum*, *O.brunneum*, *O.coerulescens* and *S.sanguineum*) were not detected again in the watercourse. Several species were vulnerable and protected amongst the disappeared larvae (*A.isoceles*, *S.metallica*, *L.fulva* and *O.brunneum*). In the recent investigation, the most species-rich sites, based on larvae (S = 4), were located in the lower section of the watercourse.

## Discussion

As small lowland watercourses are distinctive waterbodies of the Pannonian Lowland, thus research focused on them has been confined to this region (Dévai 1976, Dévai 1992, Dévai 1997, Dukay 2000, Nagy et al. 2004, Dévai et al. 2005, Wittner and Takács 2005). These watercourses are strongly affected by anthropogenic activities and climatic extremities (Buczyński et al. 2016, Maynou et al. 2017, B-Béres et al. 2019, Nagy et al. 2019, Somlyai et al. 2019). Their water is used mainly for water storage, irrigation and fishpond filling (Konecsny 2003, Bardóczyné 2010, Dövényi 2010, Fehér 2012). Although these water abstractions meet important agricultural and social needs, their extent is often excessive (Fehér 2012), inducing dry-outs. Additionally, lock (dam) installation, the prevalent method for water abstraction point creation (Fehér 2012), contributes to the transition of habitats located in the dammed sections towards the standing water characteristics (Varga 2004). Reservoirs created along them hold back water in high water-level period, but do not ensure the necessary water supply for watercourses in low water level periods (Bardóczyné 2010). Moreover, their low water discharge and proximity to agricultural fields and settlements make them vulnerable to organic matter pollution (Fehér 2012, Somlyai et al. 2019). The Carpathian Basin’s harsh and changeable climate further amplifies these negative impacts (Mika et al. 1995, Somlyódi 2008, Váradi 2021, Sümegi 2022, Szarka 2022).

As a result of these factors, the upper section of the studied watercourses within the Pannonian Lowland, except Ölyvös, has dried up and their middle (and occasionally, lower as well) section has become intermittent. This has led to a significant decline in Odonata diversity throughout the last decades. Disappearance of species requiring permanent water coverage and moderate flow velocity indicates that alteration of the watercourses was the main cause of this diversity loss. Bush et al. (2013) suggested that increased drought frequency favours dominant, migratory (r-strategist) species with shorter life cycles, which can easily recolonise habitats. This is confirmed by this study, as most remaining species were generalists, common, drought-tolerant or possess high dispersal abilities.

Despite the degradation, a still diverse Odonata assemblage (27 species) persisted along the watercourses in 2019 and 2021. Amongst the three watercourses, Konyári-Kálló showed the lowest diversity, while Ölyvös and Kállai-főfolyás had similar values. Kállai-főfolyás’ habitat alterations ensuring longer water coverage and moderate flow velocity for its middle section (Kf3) explain the prevalence of specialist species in it. The middle section of the watercourses showed the highest diversity due to its species-richness and presence of restricted, rare species. The lower section, although still having permanent water coverage, also experienced significant fauna reductions. It was dominated by common and tolerant species, as sensitive species rather occurred in the intermittent middle and upper sections. This supports the findings of Buczyński et al. (2016), that habitat requirements of rheophilic and specialist species are complex and extend beyond water coverage. While permanent and intermittent sites displayed no significant diversity differences, more sensitive and rare species were exclusive to the latter.

Comparison of recent and former Odonata fauna of Konyári-Kálló showed higher habitat degradation, with nearly 50% diversity loss over a decade. Despite recent surveys being carried out within one year, in contrast to the previous collections spanning four years, intensity and frequency of the current investigation ensured the detection of all species living along the watercourse. Studies of Datry et al. (2014), Piano et al. (2017) and B-Béres et al. (2019)revealed the prevalence of generalist and pioneer invertebrate and diatom taxa in intermittent watercourses. Our results support this, as relative frequencies of species indicated a shift from a balanced assemblage to an assemblage dominated by two common and pioneer species.

Although diverse species assemblages still live along the three studied watercourses, mostly adults could be found. Only 59 larvae and exuviae of 13 species were recorded in 2021. However, their presence is crucial to prove that observed adults developed in these watercourses. Larvae of some species have adapted to survive in intermittent watercourses, as they have quick development and a multivoltine life cycle, for example, species of *Ischnura* and *Coenagrion* (Valtonen 1986, Cham 1992) or drought tolerant larvae, for example, species of *Orthetrum* genus (Suhling et al. 2003). However, larvae and exuviae without adaptations also occurred in intermittent sections, raising questions about their survival. Extremely low numbers of larvae and exuviae at sampling sites suggest that observed adults were rather “guest adults” from other areas, trying to recolonise the watercourses rather than developing locally (Michiels and Dhondt 1991, Conrad et al. 1999).

Several Odonata adults have to disperse from their breeding sites and colonise new habitats due to their territorial behaviour. This dispersal is usually directed away from the breeding place rather than towards a specific destination (Johnson 1960, Johnson 1966, Anholt 1990, Michiels and Dhondt 1991, Conrad et al. 1999). Dragonflies have high dispersal capacity. Therefore, distances between breeding sites do not necessarily mean a barrier for them (Conrad et al. 1999, Angelibert and Giani 2003, Suhling et al. 2004, Bush et al. 2013), but habitat fragmentation induced by climate change increases their mortality during dispersal (Maynou et al. 2017). Dragonflies often use small secondary biotopes, such as small lowland watercourses, as stepping stones during their dispersal (Dolny et al. 2007, Harabis and Dolny 2012, Maynou et al. 2017). To detect their suitable egg-laying site, adult dragonflies use visual clues. They react to the horizontal polarization of light reflected from the surface of water (Corbet 1999) and avoid dried sections of watercourses (Buchwald 1992, Wildermuth and Horváth 2005, Hardersen 2008, Yalles Satha and Samraoui 2017). However, intermittent watercourses can give false signals to dragonflies, forming an "ecological trap” for them (Schlaepfer et al. 2002, Robertson and Hutto 2006, Hardersen 2008). Thus, adults show reproductive activities in vain; if their larvae do not have specific adaptations to droughts, those cannot complete their development (Carpenter et al. 1992, Williams 1997, Hardersen 2008, Hassall and Thompson 2008). Such ecological traps contribute to the disappearance of Odonata populations (Schlaepfer et al. 2002).

A significant portion of Odonata larvae in lowland watercourses experience regular mortality due to the intermittent nature of these watercourses. Guest assemblages still consist of a relatively large number of individuals of many species, making possible the recolonisation of periodically drying watercourses from adjacent waterbodies. However, this process does not work endlessly. Prolonged drying out of waterbodies will lead to a rapid decline in the diversity of Odonata assemblages and an increasing degradation of waterbodies.

Odonata fauna of small lowland watercourses is poorly known since most researchers are focusing mainly on mountainous or larger watercourses. This study demonstrated that small lowland watercourses have very unique Odonata assemblages and provide important services as stepping stones and secondary habitats. Since these watercourses are affected by anthropogenic disturbances and climate change, they dry out more often and for longer periods. In order to conserve their natural value and diverse Odonata assemblages, immediate actions, regulations and further investigations are needed.

## Acknowledgements

Work of IG was supported by Ministry of Innovation and Technology of Hungary, from the National Research, Development and Innovation Fund (TKP2021-NKTA). The research was carried out within the framework of the Széchenyi Plan Plus programme (RRF2.3.121202200008).

## Supplementary Material

DF57F1A7-1755-5E1F-B01A-0929D3C32407 10.3897/BDJ.11.e107919.suppl1 Supplementary material 1 Checklist of the dragonfly fauna of the investigated three small lowland watercourses Data type occurrences, abundances Brief description Checklist of the dragonfly fauna of the investigated three small lowland watercourses (Kf = Kállai-főfolyás, KK = Konyári-Kálló, Ö = Ölyvös), in the 14 sampling sites in the past (= 1968-1970, 1985-1987, 2003, 2008-2009, 2011-2012) and in 2021 with the abundances of the observed/caught species. File: oo_862391.xlsx https://binary.pensoft.net/file/862391 Kálmán Szanyi, István Grigorszky, László J Szabó, György Dévai

## Figures and Tables

**Table 1.** Characteristics of the three studied watercourses of the Pannonian Lowland.

**Watercourse**	**Kállai-főfolyás (Kf)**	**Konyári-Kálló (KK)**	**Ölyvös (Ö)**
**Region**	Nyírség	Nyírség, Berettyó–Körös-vidék	Berettyó–Körös-vidék
**Length (km)**	55	83.5	49
**Catchment area (km^2^)**	426	476	258
**Sampling sites**	Kf1-Kf4	KK1-KK7	Ö1-Ö3

**Table 2.** Characteristics of the studied sampling sites. Data about previous samplings can be found in Dévai and Miskolczi (1999) – 1983-1986; Dévai and Miskolczi (2009) – 2003; Dévai and Miskolczi (2011) – 1968-1970, 1985–1987; Viski et al. (2013) – 2008-2009, 2011-2012; Dévai et al. (2021) – 2019.

**Sampling site**	**Township**	**GPS (N/E)**	**Altitude zone**	**Years of previous samplings**	**Years of recent samplings**	**Water coverage permanency**
**Kf1**	Szakoly	47°45'57.01"N, 21°54'59.32"E	Upper section	2003	2019, 2021	Completely dried
**Kf2**	Nagykálló	47°51'5.66"N, 21°51'48.43"E	Middle section	2003	2019, 2021	Intermittent
**Kf3**	Nyíregyháza-Oros	47°57'32.49"N, 21°48'40.82"E	Middle section	1968- 1970, 1985-1987, 2003	2019, 2021	Intermittent
**Kf4**	Kemecse	48°4'2.29"N, 21°48'38.13"E	Lower section	2003	2019, 2021	Permanent
**KK1**	Nyírlugos	47°39'18.95"N, 22°01'49.50"E	Upper section	2008-2009, 2011-2012	2021	Completely dried
**KK2**	Nyírábrány	47°33'24.84"N, 22°00'38.30"E	Upper section	2008-2009, 2011-2012	2021	Completely dried
**KK3**	Bagamér	47°28'31.99"N, 21°57'55.52"E	Middle section	2008-2009, 2011-2012	2021	Intermittent
**KK4**	Létavértes	47°23'59.08"N, 21°53'27.39"E	Middle section	2008-2009, 2011-2012	2021	Intermittent
**KK5**	Hosszúpályi	47°21'29.57"N, 21°44'54.34"E	Middle section	2008-2009, 2011-2012	2021	Intermittent
**KK6**	Konyár	47°19'13.43"N, 21°40'46.69"E	Lower section	2008-2009, 2011-2012	2021	Permanent
**KK7**	Tépe	47°17'06.75"N, 21°33'12.06"E	Lower section	2008-2009, 2011-2012	2021	Permanent
**Ö1**	Bojt	47°10'20.44"N, 21°43'29.04"E	Upper section	1983-1986	2021	Intermittent
**Ö2**	Mezőpeterd	47°9'42.53"N, 21°38'20.71"E	Middle section	1983-1986	2021	Intermittent
**Ö3**	Berettyóújfalu	47°9'1.24"N, 21°33'17.33"E	Lower section	1983-1986	2021	Permanent

**Table 3.** Checklist of the Odonata fauna of the three small lowland watercourses (Kf = Kállai-főfolyás, KK = Konyári-Kálló, Ö = Ölyvös). *= protected and vulnerable species in Hungary according to Jakab (2013) and Ambrus et al. (2018). SUM = number of watercourses in which the species occurred (1-3). For details of previous and current samplings, see Table 2.

**Species**	**Previous occurrence**				**Current occurrence**			
	**Kf**	**KK**	**Ö**	**SUM**	**KF**	**KK**	**Ö**	**SUM**
*Chalcolestesparvidens* (Artobolevskij, 1929)	0	1	0	**1**	0	0	1	**2**
*Lestesbarbarus* (Fabricius, 1798)	1	1	1	**3**	1	1	1	**3**
*Lestessponsa* (Hansemann, 1823)	0	1	1	**2**	0	0	0	**0**
*Lestesvirens* (Charpentier, 1825)	0	1	0	**1**	0	0	0	**0**
*Sympecmafusca* (Vander Linden, 1820)	0	1	1	**2**	1	1	1	**3**
*Calopteryxsplendens* (Harris, 1782)	1	1	1	**3**	1	1	1	**3**
*Platycnemispennipes* (Pallas, 1771)	1	1	1	**3**	1	1	1	**3**
*Coenagrionornatum* (Selys, 1850)*	1	1	0	**2**	0	0	0	**0**
*Coenagrionpuella* (Linnaeus, 1758)	1	1	1	**3**	1	1	1	**3**
*Coenagrionpulchellum* (Vander Linden, 1825)	1	1	1	**3**	1	1	1	**3**
*Enallagmacyathigerum* (Charpentier, 1840)	0	1	0	**1**	0	0	0	**0**
*Erythrommanajas* (Hansemann, 1823)	0	0	0	**0**	1	0	0	**1**
*Erythrommaviridulum* (Charpentier, 1840)	1	1	1	**3**	1	1	1	**3**
*Ischnuraelegans* (Vander Linden, 1820)	1	1	1	**3**	1	1	1	**3**
*Ischnurapumilio* (Charpentier, 1825)	0	1	1	**2**	1	1	1	**3**
*Aeshnaaffinis* Vander Linden, 1820	0	1	1	**2**	1	1	1	**3**
*Aeshnamixta* Latreille, 1805	0	1	1	**2**	1	0	0	**1**
*Aeshnaisoceles* (Muller, 1767)*	0	1	1	**2**	1	1	0	**2**
*Anaximperator* Leach, 1815	1	1	1	**3**	1	0	1	**2**
*Brachytronpratense* (Muller, 1764)	0	1	1	**2**	1	0	1	**2**
*Gomphusvulgatissimus* (Linnaeus, 1758)*	1	1	0	**2**	0	0	0	**0**
*Somatochloraflavomaculata* (Vander Linden, 1825)*	1	0	1	**2**	1	0	0	**1**
*Somatochlorameridionalis* Nielsen, 1935	0	1	1	**2**	0	1	0	**1**
*Somatochlorametallica* (Vander Linden, 1825)*	0	1	0	**1**	0	0	0	**0**
*Crocothemiserythraea* (Brullé, 1832)	1	0	0	**1**	1	0	0	**1**
*Libelluladepressa* Linnaeus, 1758	1	1	1	**3**	1	1	1	**3**
*Libellulafulva* Muller, 1764*	0	1	1	**2**	1	1	1	**3**
*Libellulaquadrimaculata* Linnaeus, 1758	0	1	0	**1**	0	0	0	**0**
*Orthetrumalbistylum* (Selys, 1848)	1	1	0	**2**	1	1	1	**3**
*Orthetrumbrunneum* (Fonscolombe, 1837)*	0	1	0	**1**	1	0	1	**2**
*Orthetrumcancellatum* (Linnaeus, 1758)	0	1	1	**2**	0	0	0	**0**
*Orthetrumcoerulescens* (Fabricius, 1798)	1	1	1	**3**	1	0	0	**1**
*Sympetrumdepressiusculum* (Selys, 1841)*	0	1	0	**1**	0	0	0	**0**
*Sympetrumflaveolum* (Linnaeus, 1758)	1	0	1	**2**	0	0	0	**0**
*Sympetrummeridionale* (Selys, 1841)	1	1	1	**3**	1	1	1	**3**
*Sympetrumpedemontanum* (Allioni, 1766)*	0	0	1	**1**	0	0	0	**0**
*Sympetrumsanguineum* (Muller, 1764)	1	1	1	**3**	1	1	1	**3**
*Sympetrumstriolatum* (Charpentier, 1840)	1	1	1	**3**	1	0	1	**2**
*Sympetrumvulgatum* (Linnaeus, 1758)	1	1	1	**3**	0	0	0	**0**
**Total number of species**	20	34	27	**38**	25	17	20	**27**

**Table 4.** Number of caught species (S) and individuals (N), values of Odonata Conservation (OCI) and Standardised Odonata Conservation Indices (OCI’), values of Shannon-Wiener (SW) and Simpson diversity indices by studied sampling sites and total samples of three watercourses in 2021, considering their altitude zonation (upper section, middle section, lower section) and water coverage permanency (dried, intermittent, permanent).

	**S**	**N**	**OCI**	**OCI**'	**Simpson**	**SW**
**KK1**	0	0	0	0	0	0
**KK2**	0	0	0	0	0	0
**KK3**	6	45	2.08	0.35	0.72	1.46
**KK4**	9	97	3.50	0.39	0.67	1.48
**KK5**	6	51	2.50	0.42	0.60	1.18
**KK6**	11	87	4.08	0.37	0.78	1.95
**KK7**	9	44	3.33	0.37	0.71	1.63
**Kf1**	0	0	0	0	0	0
**Kf2**	14	78	6.17	0.44	0.79	2.03
**Kf3**	20	124	8.67	0.43	0.85	2.43
**Kf4**	10	63	4.08	0.41	0.75	1.79
**Ö1**	12	70	5.17	0.43	0.84	2.17
**Ö2**	8	63	3.17	0.40	0.81	1.84
**Ö3**	14	89	5.50	0.39	0.88	2.36
**Kf (total)**	22	265	9.92	0.45	0.81	2.32
**KK (total)**	17	324	6.92	0.41	0.71	1.80
**Ö (total)**	20	222	8.58	0.43	0.87	2.50
**Upper s**	12	70	5.17	0.43	0.84	2.17
**Middle s**	24	458	10.92	0.45	0.78	2.18
**Lower s**	17	283	7.00	0.41	0.82	2.22
**Dried**	0	0	0	0	0	0
**Intermittent**	22	404	10.00	0.45	0.77	2.11
**Permanent**	21	407	9.25	0.44	0.83	2.32

**Table 5.** Number of species and individuals found in larval or exuvial form at the studied sites with intermittent and permanent water coverage, in 2021. L = larval form, E = exuvial form.

**Species**	**Intermittent**	**Permanent**	**Form**
*Calopteryxsplendens*	0	1	L
*Coenagrionpuella*	10	8	L
*Coenagrionpulchellum*	0	1	L
*Ischnuraelegans*	5	10	L
*Ischnurapumilio*	0	3	L
*Anaximperator*	3	2	L
*Somatochlorameridionalis*	1	0	E
*Libelluladepressa*	6	0	L
*Orthetrumalbistylus*	1	0	L
*Orthetrumcoerulescens*	1	0	L
*Sympetrummeridionale*	0	1	L
*Sympetrumsanguineum*	0	1	L
*Sympetrumstriolatum*	0	5	L
**Total number of species**	7	9	
**Total number of individuals**	27	32	

**Table 6.** Number of species (S), values of Simpson and Shannon-Wiener (SW) diversity indices of the studied sampling sites of Konyári-Kálló small lowland watercourse between the years of 2008-2009, 2011-2012 (= past) and 2021 (= present). The seven most common species (Common sp.) in the past (2008-2009, 2012-2013) and present (2021) can be also found with their relative frequencies (RF%).

**Past**								
	**KK1**	**KK2**	**KK3**	**KK4**	**KK5**	**KK6**	**KK7**	**SUM**
**S**	10	17	18	20	26	15	13	34
**Simpson**	0.759	0.881	0.923	0.925	0.854	0.832	0.847	0.920
**SW**	1.716	2.444	2.717	2.756	2.345	2.228	2.170	2.799
**Common sp.**	*C.splend*	*L.fulva*	*I.elega*	*S.sangu*	*C.puell*	*C.pulch*	*S.fusca*	
**RF**%	13.40	12.64	11.20	10.11	8.85	6.91	6.15	
**Present**								
	**KK1**	**KK2**	**KK3**	**KK4**	**KK5**	**KK6**	**KK7**	**SUM**
**S**	0	0	6	9	6	11	9	17
**Simpson**	0	0	0.716	0.668	0.596	0.781	0.705	0.712
**SW**	0	0	1.461	1.478	1.178	1.945	1.634	1.803
**Common sp.**	*C.puell*	*S.sangu*	*S.merid*	*L.barba*	*P.penni*	*C.pulch*	*I.elega*	
**RF**%	49.07	19.44	6.17	5.56	3.09	2.78	2.78	

**Table 7.** Number of species which were found as larvae or exuviae at the sampling sites located in upper, middle and lower sections of the Konyári-Kálló small lowland watercourse in the past (= 2008-2009, 2011-2012) and in the present (= 2021).

**Species**	**Past**				**Present**			
	**Upper**	**Middle**	**Lower**	**Sum**	**Upper**	**Middle**	**Lower**	**Sum**
*Chalcolestesparvidens*	1	1	0	**1**	0	0	0	**0**
*Lestesbarbarus*	1	0	0	**1**	0	0	0	**0**
*Sympecmafusca*	1	1	1	**1**	0	0	0	**0**
*Calopteryxsplendens*	0	1	0	**1**	0	0	0	**0**
*Platycnemispennipes*	1	1	0	**1**	0	0	0	**0**
*Coenagrionpuella*	0	0	0	**0**	0	1	1	**1**
*Coenagrionpulchellum*	0	1	1	**1**	0	0	1	**1**
*Erythrommaviridulum*	0	0	1	**1**	0	0	0	**0**
*Ischnuraelegans*	1	1	1	**1**	0	0	1	**1**
*Ischnurapumilio*	0	0	0	**0**	0	0	1	**1**
*Aeshnamixta*	0	1	1	**1**	0	0	0	**0**
*Anaciaeschnaisosceles*	0	0	1	**1**	0	0	0	**0**
*Somatochlorameridionale*	0	0	0	**0**	0	1	0	**1**
*Somatochlorametallica*	1	1	0	**1**	0	0	0	**0**
*Libelluladepressa*	1	0	0	**1**	0	1	0	**1**
*Libellulafulva*	1	1	1	**1**	0	0	0	**0**
*Orthetrumalbistylum*	1	1	0	**1**	0	0	0	**0**
*Orthetrumbrunneum*	1	0	0	**1**	0	0	0	**0**
*Orthetrumcoerulescens*	1	0	1	**1**	0	0	0	**0**
*Sympetrumsanguineum*	1	1	1	**1**	0	0	0	**0**
**Total number of species**	12	11	9	**17**	0	3	4	**6**
